# Traumatic external jugular vein aneurysm after a kendo thrust (tsuki)

**DOI:** 10.1016/j.jvscit.2026.102498

**Published:** 2026-09-04

**Authors:** Shin Sugasawa, Noriyuki Yahagi, Shingo Ohno, Tatsuo Masubuchi, Masaaki Suzuki, Junichiro Kumai

**Affiliations:** a Department of Neurosurgery, Chiba-Nishi General Hospital, Chiba, Japan; b Department of Head and Neck Surgery, Chiba-Nishi General Hospital, Chiba, Japan; c Department of Diagnostic Pathology, Chiba-Nishi General Hospital, Chiba, Japan

**Keywords:** External jugular vein aneurysm, Venous aneurysm, Trauma, Kendo, Vascular surgery

## Abstract

Aneurysm of the external jugular vein is rare, and a traumatic case with a documented mechanism and pathological findings is exceptional. A woman in her 50s sustained a blunt injury to the left anterior neck from a kendo thrust (tsuki). One month later she developed a left cervical swelling. Ultrasound study and computed tomography showed a 2-cm saccular dilatation with a cranial filling defect, indicating partial thrombosis. After 4 months of observation, the aneurysm was resected at the patient’s request. Histopathology showed segmental loss of elastic fibers and adventitial rupture without organized thrombus. Recognizing venous injury after a kendo thrust enables timely diagnosis.

Sports injuries are an important part of trauma care. In kendo (Japanese fencing), protective equipment (bogu) shields most of the head and neck, but the thrusting technique (tsuki) can deliver a direct force to the anterior neck. Reported tsuki-related injuries include common carotid artery dissection^1^ and pneumomediastinum from tracheal injury,^2^ whereas venous injury of the neck is rarely described. Aneurysms of the external jugular vein (EJVA) are themselves uncommon and are usually idiopathic or congenital; a traumatic EJVA is rare.^3^^,^^4^ To our knowledge, no previous report has documented both the mechanism of injury and the histopathological findings. We report a traumatic EJVA that developed after a kendo tsuki, with histopathologic correlation. The patient provided written informed consent for the publication of this report and the accompanying images.

## Case report

A woman in her 50s was struck on the left anterior neck during a kendo match when the bamboo sword (shinai) passed through the gap between the throat protector (tsuki-dare) and the facemask flap (men-dare). About 1 month later she noticed swelling of the left lateral neck and was referred to our department for evaluation. Her medical and family histories were unremarkable. Swelling was her only symptom; she had no pain, tenderness, dysphagia, hoarseness, or neurologic deficit. The mass was soft and nonpulsatile and enlarged with a Valsalva maneuver.

Initial color Doppler ultrasound study showed a hypoechoic solid mass in continuity with the EJVA. No color flow was detected within the mass, whereas venous flow was preserved in the nonaneurysmal EJVA (Fig 1, *A*). Contrast-enhanced computed tomography, performed the same day, demonstrated a saccular dilatation of the EJVA, approximately 2 cm in maximum diameter, with a filling defect at the cranial aspect (Fig 1, *B-E*). These findings were interpreted as partial thrombosis with a residual patent lumen; the absence of color flow probably reflected velocities below the detection threshold of color Doppler, consistent with enlargement of the mass during a Valsalva maneuver. A traumatic EJVA was diagnosed from these findings and the mechanism of injury.

**Fig 1:**
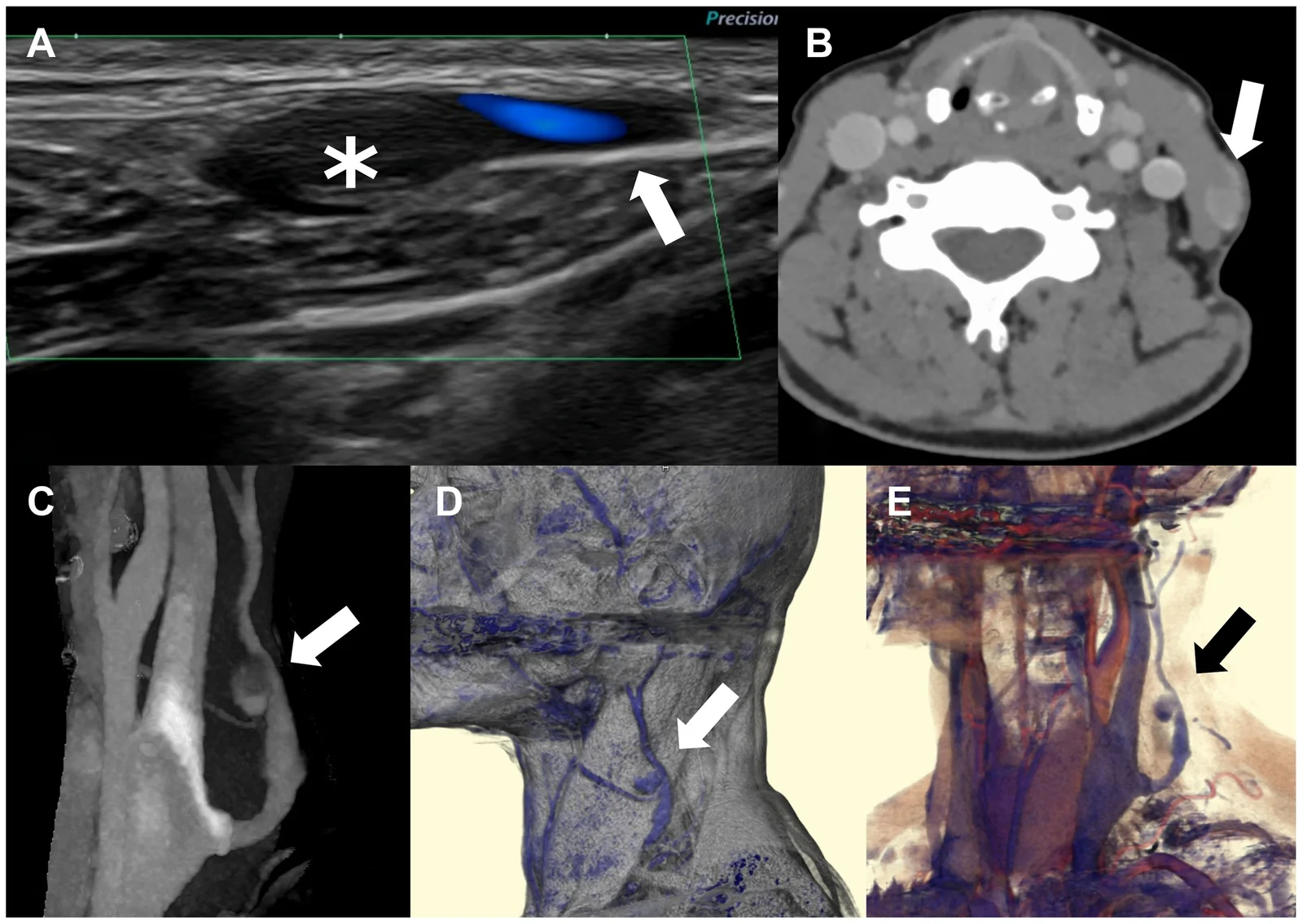
Preoperative imaging. **A,** Color Doppler ultrasound study: a hypoechoic solid mass in continuity with the external jugular vein (*asterisk*). No color flow is detected within the mass, whereas venous flow is preserved in the nonaneurysmal external jugular vein (*arrow*). **B,** Contrast-enhanced computed tomography (CT), axial view: a faintly enhancing saccular structure in the left subcutaneous neck (*white arrow*). **C,** Contrast-enhanced CT, maximum-intensity projection: saccular dilatation continuous with the external jugular vein (*white arrow*); a filling defect is present at its cranial aspect. **D,** Three-dimensional CT reconstruction (venous): dilatation along the course of the left external jugular vein (*white arrow*). **E,** Three-dimensional CT reconstruction (arteriovenous fusion; arteries *red*, veins *blue*): dilatation of the left external jugular vein (*black arrow*).

As thrombus was suspected within the aneurysm, anticoagulation was started empirically at diagnosis; as no regimen has been established for this condition, edoxaban 30 mg once daily (reduced dose for body weight ≤ 60 kg) was given, extrapolated from dosing for venous thromboembolism. The indication was reassessed 1 week later: the patient had remained asymptomatic, and superficial vein thrombosis at other sites carries a low risk of propagation and pulmonary embolism. Anticoagulation was therefore discontinued, and the patient was followed as an outpatient without antithrombotic therapy. No thromboembolic event occurred during the 4 months between diagnosis and resection. The patient requested treatment because of her concern about embolization and for cosmetic reasons, and the aneurysm was resected.

A head and neck surgeon assisted with positioning and exposure. Under general anesthesia, sustained positive airway pressure (a Valsalva-like maneuver) was applied to distend the aneurysm and aid localization (Fig 2, *A*), and a transverse incision was made along a skin crease. The aneurysm was dissected circumferentially; the proximal and distal EJVA and the adjacent branches were ligated and divided, and the lesion was removed en bloc (Fig 2, *B*). The resected specimen measured 17 mm in maximum diameter (Fig 2, *C*).

**Fig 2:**
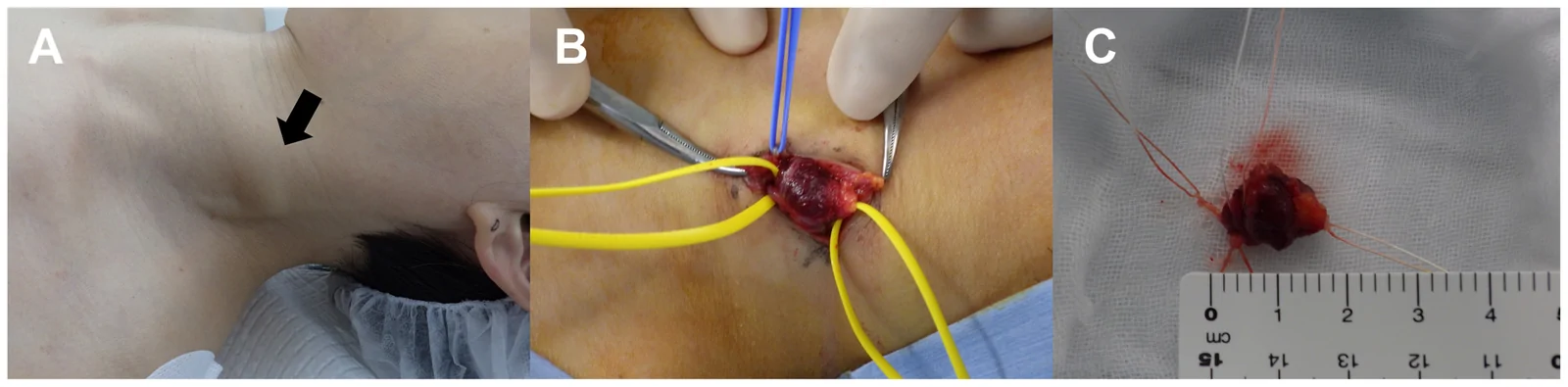
Macroscopic and intraoperative findings (*left neck*). **A,** Sustained positive airway pressure applied under general anesthesia (a Valsalva-like maneuver) distends the external jugular vein aneurysm (*black arrow*). **B,** The aneurysm has been dissected circumferentially; the proximal and distal external jugular vein are secured with yellow vessel loops and a cardiac-side branch with a blue loop. **C,** Resected specimen on a centimeter ruler; maximum diameter approximately 17 mm.

The entire specimen was sectioned serially, including the point of maximum diameter. Histopathology showed focal thinning of the venous wall to about one-third of the normal thickness, a segmental defect of elastic fibers, and rupture of the adventitia, without inflammatory cell infiltration, hemosiderin deposition, organized hematoma, or intraluminal thrombus (Fig 3). Elastica-van Gieson staining showed fragmentation and loss of elastic fibers at the ruptured and dilated segments, and Masson trichrome staining showed no fibrosis or collagen proliferation.

**Fig 3:**
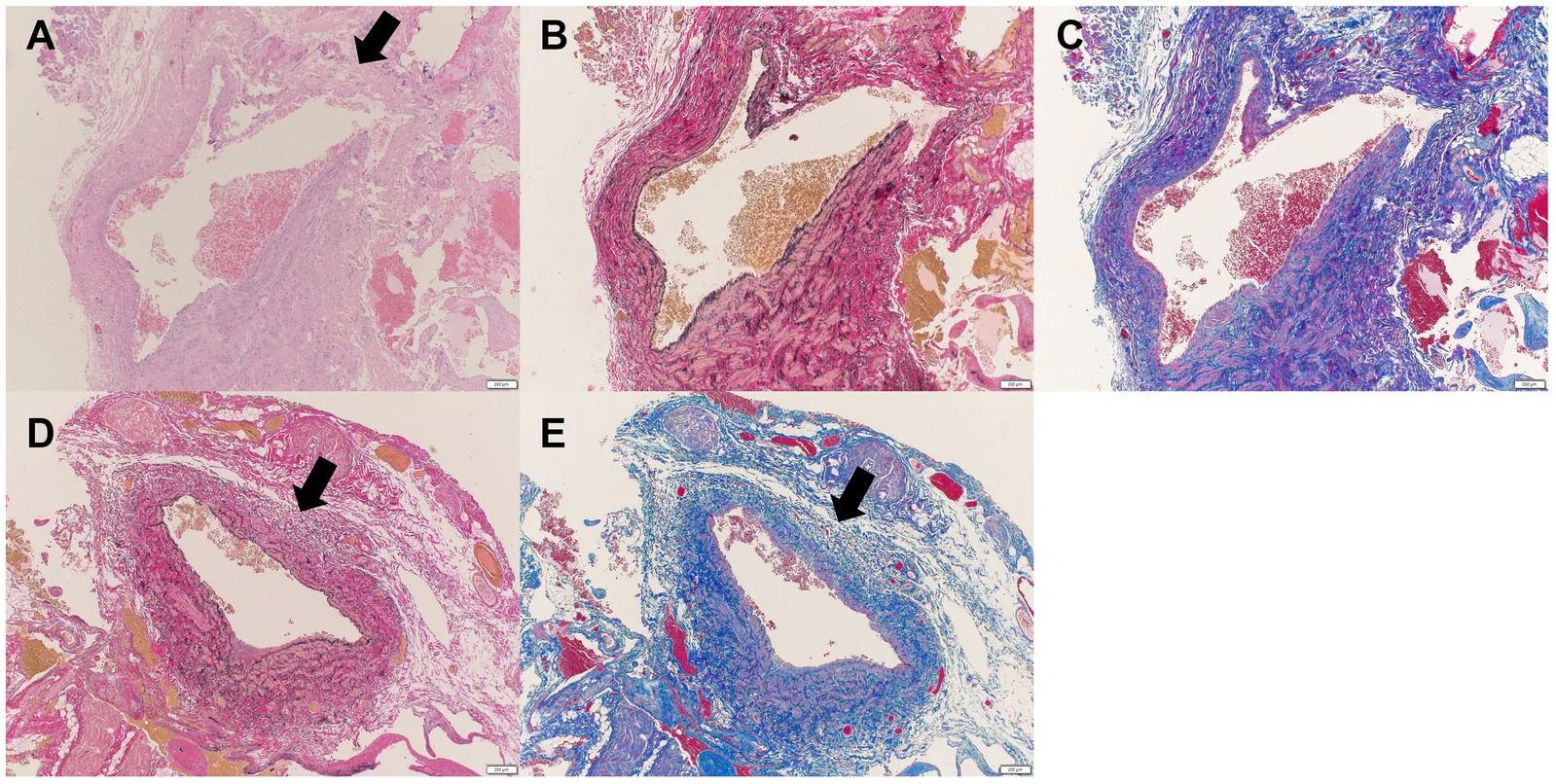
Histopathology of the resected aneurysm wall. Rupture site **(A**-**C)** and dilated segment **(D** and **E)**. **A,** Hematoxylin-eosin stain (rupture site): thinning and discontinuity of the venous wall (*arrow*); no inflammatory cell infiltration. **B** and **C,** Elastica-van Gieson stain **(B)** and Masson trichrome stain **(C)** of the same rupture site: fragmentation and loss of elastic fibers on Elastica-van Gieson, without fibrosis or collagen proliferation on Masson trichrome. **D** and **E,** Elastica-van Gieson stain **(D)** and Masson trichrome stain **(E)** of the dilated segment: the wall is thinned to about one-third of normal with reduced elastic fibers (*arrow*). Scale bars, 200 μm.

The postoperative course was uneventful. At 1 month there was no recurrence, no venous congestion, and no cosmetic complaint, and no antithrombotic therapy was needed.

## Discussion

The tsuki-dare and the men-dare do not overlap completely, and this gap in the bogu is a point of vulnerability of the anterior neck. Symptoms may be absent immediately after the injury, and the lesion may become clinically evident only weeks later, as in our patient; venous injury should therefore be considered after a tsuki even when the initial findings are unremarkable.

Venous aneurysms are classified as true aneurysms, in which the three wall layers and endothelial lining are preserved, and pseudoaneurysms, in which wall continuity is disrupted and the wall is composed of fibrous tissue or organized hematoma.^5^, ^6^, ^7^, ^8^ In our case, adventitial rupture and a segmental defect of elastic fibers were present, whereas organized hematoma, hemosiderin deposition, and inflammatory infiltration were absent. These findings indicate traumatic disruption of wall continuity but differ from a typical chronic pseudoaneurysm, and likely reflect a localized traumatic wall injury.^8^ Together with the clear mechanism of injury, they support a traumatic venous aneurysm. The EJVA is a superficial, thin-walled vessel that lies immediately beneath the skin and platysma and is relatively unsupported, which may predispose it to focal wall disruption and subsequent aneurysmal dilatation after a direct blunt impact.

Duplex ultrasound study is the initial modality of choice when a venous aneurysm is suspected; color Doppler demonstrates the communication between the aneurysm and the parent vein,^9^ and cross-sectional imaging then defines the extent of the lesion and its relationship to surrounding structures.

The differential diagnosis of a lateral neck mass is broad and includes lymphadenopathy, a branchial cleft or other cystic lesion, a laryngocele, and vascular lesions such as a carotid body tumor or an arteriovenous malformation. After blunt cervical trauma, a hematoma, an arterial pseudoaneurysm, and a traumatic arteriovenous fistula should also be considered. In our patient, the absence of pulsatility and bruit, enlargement of the mass with a Valsalva maneuver, and its continuity with the EJVA on duplex ultrasound study excluded these entities.

Thrombosis is the most common complication of EJVA (approximately 37% in a systematic review),^4^ and jugular venous aneurysms may rarely cause pulmonary embolism.^10^^,^^11^ In cannula-provoked upper-extremity superficial vein thrombophlebitis, anticoagulation has been reported to be unnecessary in most patients.^12^ No standard treatment has been established for a thrombosed EJVA. Our patient remained free of thromboembolic events over these 4 months; although no firm conclusion can be drawn from a single patient, nonoperative management without antithrombotic therapy appears reasonable, particularly for patients who prefer to avoid operation. On the other hand, resection with ligation is commonly performed for EJVA,^3^^,^^4^^,^^13^ and no relevant complications have been reported after ligation of the EJVA without reconstruction.^3^

Although imaging had suggested an intraluminal thrombus, none was found on histologic examination. This discrepancy likely reflects spontaneous resolution of a nonorganized thrombus during the 4-month interval between diagnosis and resection.

## Conclusions

A kendo tsuki can disrupt the wall of the EJVA and produce a traumatic venous aneurysm that becomes apparent weeks after an apparently minor injury. Adventitial rupture with segmental loss of elastic fibers distinguishes this lesion from an idiopathic venous aneurysm and supports a traumatic etiology. Both observation and resection are reasonable options; resection with ligation of the EJVA is well tolerated and is appropriate when thromboembolism or appearance is a concern.

## Funding

None.

## Patient consent

The patient provided written informed consent for the publication of this report and the accompanying images.

## Declaration of generative AI and AI-assisted technologies in the writing process

During the preparation of this work, the authors used Claude (Anthropic) for translation from Japanese and English-language editing. After using this tool, the authors reviewed and edited the content as needed and take full responsibility for the content of the publication. The tool was not used to generate scientific content, data, figures, or references, and no AI tool is listed as an author.

## Disclosures

None.
